# The influence of consumers’ knowledge on their responses to genetically modified foods

**DOI:** 10.1080/21645698.2020.1840911

**Published:** 2020-11-02

**Authors:** Hyesun Hwang, Su-Jung Nam

**Affiliations:** a Department of Consumer Science, Convergence Program for Social Innovation, College of Social Sciences, Sungkyunkwan University, Seoul, Republic of Korea; b Department of Home Economics Education, College of Education, Jeonju University, Jeonju, Republic of Korea

**Keywords:** Genetically modified foods, consumer knowledge, perceived risk, perceived benefit

## Abstract

This study examined the influence of consumers’ knowledge on their perceptions and purchase intentions toward genetically modified foods, and the implications of these consumer responses for sustainable development in the food industry. This study distinguished between objective and subjective knowledge and identified how an imbalance between the two knowledge types influenced consumers’ attitudes and purchase intentions toward genetically modified foods. Results of a multinomial regression analysis showed that consumers with higher levels of education, income, and food involvement and more exposure to negative information about genetically modified foods tended to overestimate their actual knowledge level. The overestimation group showed a higher risk perception, lower benefit perception, and lower intention to purchase genetically modified foods than other participants. Consumers with less education and higher income were more likely to underestimate their knowledge.

## Introduction

In their attempts to improve the sustainability of our society, scientists have made numerous advances in science and technology, which have significantly impacted the food industry. Technological advances have not only improved food production but also helped to increase nutritional content of food and meet consumers’ preferences for diversity.^1–3^ The ever- increasing food demand and unavoidable climate change has called for greater flexibility and innovation in crop resilience and production systems. For several decades, plant breeding methods (e.g. genetically modified organisms (GMO), genome editing) had been the choice for crop improvement programs.^4^ However, perceived risks of new technologies in the food industry are regarded as threats to consumer health as they raise multiple questions, including the nutritional quality of food, toxicity, antibiotic resistance, and allergies.^1^

Genetically modified (GM) foods have recently become a matter of public controversy owing to both the opportunities they provide to promote sustainable food production and the potential health risks associated with their consumption.^3,5^ The advantages of GM food are not only the increase in food in developing countries, but also resistance to pests, drought tolerance, and beneficial nutrients.^6–8^ Transgenic technology, a technique used to create many GM foods, is regarded as a powerful solution for resolving food crises by creating crop cultivars that are resistant to diseases and insects and enhancing the efficiency and yield of foods and grains. Such advances help solving the problem of food shortage and improve the quality of food by maintaining the freshness of fruit and vegetables. They also prevent environmental pollution by reducing the use of herbicides and insecticides and promoting the sustainability of the environment.^9^

Nonetheless, there are controversies regarding GM food on several levels, including whether it is safe, whether it should be labeled and if so how, whether agricultural biotechnology is needed to address world hunger now or in the future.^10,11,12^ However, a recent study reports that the GMO method is safe and has no cause for worry; if anything, it is safer than the traditional ways of breeding crops.^8^

Consumers are still likely to be confused about the process of purchasing and consuming GMO food. China’s Ministry of Agriculture and the science community expressed a generally positive attitude toward GM food, but the percentage of respondents that trusted the government and scientists was only 11.7 and 23.2%, respectively.^12^ According to,^13^ the number of GM foods approved by the U.S. Food and Drug Administration and offered on supermarket shelves has steadily grown, while simultaneously, public wariness about the safety of GM foods has increased.

Therefore, it is inevitable that consumers experience anxiety about GM foods^5,12^ which may in turn affect their attitudes and behaviors regarding food choice. Research suggests that consumers may experience greater anxiety about food when 1) the risk of consuming the food occurs involuntarily, regardless of their actions or choices, 2) they deem it impossible to control the risk involved with consuming the food or the risk is substantial, and 3) the food is influenced by factors seemingly beyond scientific comprehension.^14^ Furthermore, the hazard from foods originating from technologies is associated with a higher level of risk perception.^15^ Although there is ample information to enables consumers to evaluate the risks of GM foods and to exercise informed choices, factors that are critical for consumer acceptance and risk management, consumers remain relatively uninformed about the advantages and disadvantages of GM foods.^16,17^

Within this context, the current study empirically examined consumer knowledge of GM foods and its influence on consumers’ attitudes. Specifically, the study focused on the imbalance between consumers’ objective and subjective knowledge, and how it impacted their attitudes toward the risks, benefits, and purchase intentions of GM foods. One of the goals of the study was to provide insights on the knowledge structures of consumer groups for managers in today’s food industry that are focused on sustainable development. Further, determinants of the knowledge structures are investigated to understand how they contribute to consumers’ different responses to GM foods.

## Literature Review

### Consumers’ Perceived Risks and Benefits of GM Foods

Technological advances in food production are regarded as inevitable changes in today’s food industry,^18^ and a large number of novel foods or food ingredients consumed worldwide have been produced through genetic modification since the mid-1990s.^19,20^ However, most consumers remain uninformed about GM foods; thus, it is natural that they do not fully understand the scientific basis of GM technology including its potential benefits and risks.^21,22^

Previous studies of the GM technology have indicated that consumers perceive more risks than benefits.^23,24,25,26^ asserted that consumers tend to perceive multiple risks and no benefits associated with GM foods. Consumers’ general skepticism toward GM foods is influenced by their attitudes toward GMOs.^25^oncluded that the more people are aware of GMOs, the more they perceive the benefits as being greater than the risks; nevertheless, they are generally unaware of whether or not they consume GM foods. This result is consistent with those from multiple other studies,^27–31^ which found that despite consumers’ greater understanding of GMOs and their expected benefits, they nevertheless maintain a negative attitude toward GM foods.

### Consumers’ Objective and Subjective Knowledge

Consumers’ knowledge influences their attitudes toward GM foods and other consumer goods. Previous studies indicated a positive relation between consumers’ knowledge of GM technology and their attitude toward GM foods.^29,32–35^ Consumer knowledge about GM technology is also related to consumers’ perception of the benefits and risks of GM foods^9^ and regarded as a significant factor for adjusting biased perceptions and intentions regarding GM foods.^34^ When consumers have a high level of involvement in a certain product category that is an area of personal interest for them,^36^ their product knowledge increases.^37–39^ Further, increased consumer knowledge typically raises the likelihood of searching for new information as part of the decision-making process within the product category, which in turn raises the level of consumer knowledge. Individuals are often concerned about unfamiliar hazards when they lack sufficient knowledge to anticipate or avoid negative impacts. Therefore, increased consumer knowledge regarding GM technology, and thus increased consumer information-seeking, may help shape attitudes toward GM foods.

Consumer knowledge is divided into objective and subjective knowledge because of the difference between what consumers think they know about something and what they actually know. This difference might influence consumers’ attitudes and purchase intention toward GM foods.^2,9^ Objective knowledge, namely correct information about products that are accumulated in consumers’ long-term memories (i.e. a consumer’s actual knowledge), has close relations with the ability to perform product-related tasks.^40^ By contrast, subjective knowledge is based on direct experience by consumers and their interpretation of these experiences and suggests an intimacy with products and self-selection in product engagement.^41^

Although objective and subjective knowledge are interconnected, previous studies have shown that objective knowledge is rarely the same as subjective knowledge.,^42^reported that subjective and objective knowledge, while correlated, cannot be substituted and should be measured separately. Some studies have showed that these two constructs have a weak or moderate relationship.^9,43–45^ According to the Dunning-Kruger effect,^46^ despite having little objective knowledge, consumers may think they have sufficient knowledge; moreover, although their level of objective knowledge may be high, they may sometimes evaluate their subjective knowledge as being low. Therefore, measuring the two constructs separately and identifying the imbalance between them will help identify which type of knowledge influences consumers’ responses toward GM foods, thereby providing meaningful insights to the food industry.

### Consumers’ Knowledge, Attitude, and Purchase Intention

Factors that influence consumers’ concerns or anxiety about GM foods include their level of understanding and attitudes toward the perceived risks and benefits of GM technology.^26^ Because risk and benefit perceptions are based on an individual’s evaluation of product attributes,^9,27^ they can be affected by increasing consumers’ knowledge of GM foods. When information about GM technology is lacking, consumers cannot evaluate the possible risks and benefits objectively. Therefore, the association between consumer knowledge and attitude toward GM foods based on the perceived risks and benefits needs to be examined.

Further, although it is necessary to develop GM foods, such foods lose value if consumers are anxious about their safety. Therefore, this study sought to comprehend the level of factors influencing consumers’ anxiety about GM foods and prepare countermeasures. Previous studies have shown that consumers have heard about GM foods but lack specific information about them and are largely influenced by mass media, which stresses the negative aspects of GM foods.^26,47–49^ Therefore, it is necessary to evaluate the relationship between consumers’ knowledge of GM foods and their attitudes and behaviors, which will help to ensure consumers’ safety and suggest managerial insights for the food industry.

Accordingly, this study measures consumers’ subjective and objective knowledge of GM foods to represent their attitudes and purchase intentions. Based on their levels of subjective and objective knowledge, consumers were categorized into knowledge groups. If a consumer’s objective knowledge was the same as their subjective knowledge, they were unlikely to raise issues about GM foods. However, if there was an imbalance between subjective and objective knowledge, i.e. if consumers tended to under- or overestimate their knowledge level, there was a likelihood for the imbalance to lead to non-ideal or unsuitable purchase decision-making behavior due to the lack of proper information. Therefore, this study divided consumers into knowledge groups according to their levels of subjective and objective knowledge of GM foods. In this respect, this study can provide basic information to the food industry to allow for the construction of an integrated perspective on today’s technological advances regarding GM foods, which is based on the perceived risks and benefits and purchase intentions of the knowledge groups.

The research questions for this study are as follows. First, how are consumers distributed according to their subjective and objective knowledge? Second, which factors influence the classification of participants into these knowledge groups? Third, is there a difference between perceived risk, perceived benefit, and purchase intention related to GM foods according to consumers’ knowledge groups?

## Methods

### Data

This study used the 2014 Survey on Consumers’ Perception of GMOs conducted by the National Institute of Agricultural Sciences, part of the Rural Development Administration in Korea. Responses were collected through a self-administered survey, from June 13 to 30, 2014, using a structured questionnaire intended for 1,000 adults in Korea. This study employed a secondary data analysis method and conformed to ethical standards. In the national survey, preliminary instructions were sent to the participants, and research cooperation was obtained. The sampled population was based on the resident registration population and was selected using quota sampling in consideration of district, gender, and age. Table 1 presents the participants’ characteristics.

**Table 1.** Characteristics of participants

Characteristics	*n* (%)
Gender	
Male	493 (49.3)
Female	507 (50.7)
Age	
20–29	168 (16.8)
30–39	206 (20.6)
40–49	222 (22.2)
50–59	169 (19.6)
60–69	165 (19.5)
70 and over	13 (1.3)
Education	
No education	2 (0.2)
Elementary school	47 (4.7)
Middle school	81 (8.1)
High school	345 (34.5)
College	512 (51.2)
Graduate	13 (1.3)
Monthly income^a^	
<855.62	10 (1.0)
855–1712	71 (7.1)
1713–2569	135 (13.5)
2570–3425	299 (29.9)
3426–4282	269 (26.9)
4283–5138	148 (14.8)
5139–5995	48 (4.8)
5995<	20 (2.0)
Media dependency	
Depending on offline media	819 (82.2)
Depending on online media	177 (17.8)

*Note: N = *1,000. ^a^The unit is USD

Respondents comprised 493 men (49.3%) and 507 (50.7%) women. A total of 222 (22.2%) were in their forties, 206 (20.6%) in their thirties, and 169 (16.9%) in their fifties. Most participants were college graduates (51.2%), while 34.5% were high school graduates, and 8.1% were middle school graduates. Altogether, 299 (29.9%) consumers earned USD 2570–3425 per month and 269 (26.9%) earned USD 3425–5138 per month. With regard to media dependency, 819 (81.9%) consumers depended more on offline media, while 177 (17.8%) depended more on online media.

### Measures

The measures were developed by the National Institute of Agricultural Sciences in 2011, and they have been elaborated upon to improve their validity and reliability based on an annual survey. With regard to objective knowledge, respondents answered seven true-or-false questions on GM food-related objective facts, receiving one point for the right answer and zero points for the wrong one. The score of objective knowledge was transformed into a four-point scale to compare the mean scores of two types of knowledge. Respondents with a score above the median (i.e. 2.28 out of 4) were considered to have high objective knowledge.

With regard to subjective knowledge, a single self-report item was used.^50^ In this study, respondents answered one question, “How much do you know about GMOs and GM foods.” Possible responses were “not at all” (one point), “don’t know” (two points), “know somewhat” (three points), and “very much” (four points). According to the median (i.e. 2.0), respondents were classified into high and low subjective knowledge groups; respondents who scored three or four points were placed into the high subjective knowledge group.

The mean score of perceived risk was calculated by measuring the respondents’ agreement with three statements (“potential risk of influencing the environment is high,” “potential risk of influencing the human body is high,” and “GM foods might cause harm to the ecosystem”), using a five-point Likert scale. The mean score of perceived benefit was also calculated by measuring the respondents’ agreement with three statements (“beneficial for the environment and health because of reduced use of herbicides, insecticides, and fertilizers”; “helpful for solving the food problem because they increase yields”; and “lowers production cost and product price and improves quality”), using a five-point Likert scale. Consumer involvement in food and health was measured based on the answers to a single item for each, using a five-point Likert scale: “How much are you interested in food/health?” Purchase intention was measured based on the answers to one question, using a five-point Likert scale; the higher the score, the higher the purchase intention. GM food-related information tendency measured the overall tendency of GM food-related information that consumers gained from multiple sources to provide negative, neutral, or positive information regarding GM foods. One point was given for very negative information, two points for negative, three points for fair, four points for positive, and five points for very positive. Media dependency was measured using a multiple-choice question asking respondents to choose the one medium they have referred to most frequently to acquire information about GM foods; then, respondents were classified into online or offline media dependency, and the variable of media dependency was recoded into a binary variable (offline = 0, online = 1). The validity and reliability of the measures comprising multiple items were verified by exploratory factor analysis and item-to-total correlations as shown in Table 2. An exploratory principal components analysis was performed to examine construct validity with Varimax rotation. The Kaiser-Meyer-Olkin (KMO) was 0.711, and the approximate chi-square of the spherical Bartlett test was 2006.319 at the 0.1% significance level, which shows that the use of a factor analysis was appropriate.^51–54^ All items loaded highly ranging from.647 to .859 into three factors that corresponded with the constructs of the measurement. Results of the reliability test based on Cronbach’s alpha were .512 and .730. According to^55,56^ the value of alpha depends on the number of items on the scale; thus, item correlations need to be considered together.^57^ Therefore, the reliability test based on item-to-total correlations ranged from .633 to .847, which showed internal consistency of the measures.

**Table 2.** Factor loadings and reliability estimates for perceived risk and perceived benefit measures

	Items	Factor loadings	Item-to-total correlation	Cronbach’s α
Perceived risk	Potential risk of influencing the environment is high	.844	.821***	.512
	Potential risk of influencing the human body is high	.859	.839***	
	GM foods might cause harm to the ecosystem	.681	.762***	
Perceived benefit	GM foods are beneficial for the environment and human health because they use fewer herbicides, insecticides, and fertilizers	.647	.761***	.730
	GM foods are helpful for solving the food problem because they increase yields	.690	.633***	
	GM foods lower production cost and product price and improve quality	.762	.738***	

*Note*: ***p <.001. GM = genetically modified.

### Analysis

This study divided consumers into four groups according to their levels of subjective and objective knowledge: knowledge group (KG), ignorance group (IG), overestimation group (OG), and underestimation group (UG). KG has high subjective knowledge and high objective knowledge; IG has low subjective knowledge and low objective knowledge; OG has high subjective knowledge and low objective knowledge; and UG has low subjective knowledge and high objective knowledge. An ANOVA with the post-hoc Tukey test was conducted to examine the mean differences in the perceived risk, perceived benefit, and purchase intention of the four groups. To examine which factors affect the classification of consumers’ knowledge groups, this study employed a multinomial regression using SPSS 21.0.

## Results

### Consumer Knowledge Groups according to the Level of Objective and Subjective Knowledge

Table 3 shows the distribution of consumers into four knowledge groups according to the level of their objective and subjective knowledge. Altogether, 483 consumers (48.3%) were classified into IG, 170 consumers (17.0%) into UG, 158 consumers (15.8%) into OG, and 189 consumers (18.9%) into KG. The χ^2^ value for the group division was 79.605, which was statistically significant. Hence, IG was shown to have the highest proportion of respondents, while OG had the lowest. This result indicated that a large proportion of consumers were leaning toward low objective knowledge and about 33% had imbalanced knowledge. The mean scores of the objective and subjective knowledge of all respondents was 2.20 out of 4 and 2.13 out of 4, as shown in Table 4. When comparing the mean scores of the two types of knowledge, consumers tended to have higher objective knowledge than subjective knowledge (*t* = 2.397, *p* < .05).

**Table 3.** Distribution of participants among consumer knowledge groups

		Subjective knowledge		
		Low	High	χ^2^
Objective knowledge	Low	Ignorance Group (IG)	Overestimation Group (OG)	79.605***
		483 (48.3%)	158 (15.8%)	
	High	Underestimation Group (UG)	Knowledge Group (KG)	
		170 (17.0%)	189 (18.9%)	

*Note*: ***p <.001

**Table 4.** Objective and subjective knowledge of consumer knowledge groups

	Objective knowledge	Subjective knowledge	
Knowledge groups	Mean (SD)^a^	Mean (SD)^a^	*t*
IG	1.58 (0.71)	1.63 (0.48)	−1.577
OG	1.87(0.53)	3.04 (0.19)	−26.012***
UG	3.13 (0.38)	1.71 (0.46)	32.986***
KG	3.25 (0.43)	3.02 (0.13)	7.079***
Total	2.20 (0.95)	2.13 (0.76)	2.397*

*Notes*: *p <.05, ***p <.001. ^a^The mean scores are calculated based on a 4-point scale. IG = ignorance group; OG = overestimation group; UG = underestimation group; KG = knowledge group.

### Determinants of Consumers’ Knowledge Groups regarding GM Foods

Table 5 shows the results of the multinomial regression for examining the determinants that influenced the classification of consumers’ knowledge groups. Specifically, the independent variables included in the regression model as determinants were gender, age, education, monthly income, food and health involvement, media dependency for searching for information regarding GM foods, and information tendency related to GM foods.

**Table 5.** Multinomial regression of consumer knowledge group

	Overestimation group				Underestimation group				Knowledge group			
	*B*	SE	Exp(B)	*p*	*B*	SE	Exp(B)	*p*	*B*	SE	Exp(B)	*p*
Gender	.035	.200	1.035	.861	.252	.190	1.286	.185	−.083	.185	.921	.656
Age	−.007	.009	.993	.452	.001	.009	1.001	.945	−.006	.009	.994	.475
Education	.356	.179	1.427	.047	−.254	.143	.776	.076	.109	.155	1.116	.481
Monthly income	.320	.079	1.377	.000	.175	.078	1.192	.025	.226	.075	1.253	.003
Food involvement	.416	.156	1.516	.007	.208	.140	1.231	.137	.681	.153	1.976	.000
Health involvement	.160	.161	1.173	.321	.180	.151	1.198	.232	.220	.150	1.246	.144
Media dependency	.289	.222	1.334	.194	−.085	.236	.919	.720	.356	.209	1.427	.089
GM foods-related information tendency	−.490	.124	.613	.000	.034	.114	1.035	.765	−.015	.111	.985	.891

*Notes*: Reference group = ignorance group; −2Log likelihood = 2.336E3; Chi-square = 136.703 (*df *= 24, *p* <.000); Gender (0 = female, 1 = male); Education (0 = No education, 1 = Elementary school, 2 = Middle school, 3 = High school, 4 = College, 5 = Graduate school); Monthly income (1 = under 1 million, 2 = 1–1.99 million, 3 = 2–2.99 million, 4 = 3 − 3.99 million, 5 = 4–4.99 million, 6 = 5–5.99 million, 7 = 6–6.99 million, 8 = 7 million +); Media dependency (0 = off-line, 1 = on-line); GM foods-related information tendency are scored on a 5-point Likert scale, with responses ranging from 1 (very negative) to 5 (very positive), with a higher score corresponding to a greater positive orientation. GM = genetically modified.

There was no significant effect of gender, age, and health involvement on consumers’ knowledge groups. Education was a significant determinant of OG and UG. The more education consumers had, the more likely they were to be classified into OG (Exp(B) = 1.427, *p* < .05); a reversed tendency was shown for UG (Exp(B) = 0.776, *p* < .10). Monthly income showed a positive effect on the probability of classifying consumers into OG (Exp(B) = 1.377, *p* < .001), UG (Exp(B) = 1.192, *p* < .05), and KG (Exp(B) = 1.253, *p* < .01). The higher the monthly income of consumers, the more likely they were to be classified into OG, UG, or KG compared with IG. Food involvement showed a positive effect on the probability of OG (Exp(B) = 1.516, *p* < .01) and KG (Exp(B) = 1.976, *p* < .001) classification. According to media dependency regarding GM food-related information, when consumers depended on online media, the possibility of being classified into KG increased at the 10% significance level. Finally, with regard to GM food-related information tendency, OG consumers showed a negative value, which means that the more they were exposed to negative information on GM foods, the more likely they were to be classified into OG in comparison with IG.

### Perceived Risks, Perceived Benefits, and Purchase Intention according to Consumers’ Knowledge Groups

Table 6 shows the mean scores of perceived risk, perceived benefit, and purchase intention. The mean score of perceived risks was 3.63 and that of perceived benefits was 3.19. The mean score of intention to purchase GM foods was 2.60 and that of GM food-related information tendency was 2.67, which is relatively negative.

**Table 6.** Mean differences in the perception of risk, perception of benefits, and purchase intention by consumer knowledge group

	Perception of risk			Perception of benefits			Purchase intention		
	Mean	SD	F	Mean	SD	F	Mean	SD	F
IG	3.56^a^	.62	15.601***	3.22^b^	.58	13.033***	2.58^a^	.84	5.846***
OG	3.94^b^	.72		2.93^a^	.75		2.42^a^	.93	
UG	3.51^a^	.64		3.30^b^	.48		2.62^ab^	.92	
KG	3.66^a^	.67		3.27^b^	.60		2.81^b^	.89	

*Notes*: ***p <.001, ^a,b^ significantly difference in post-hoc Tukey test at alpha =.05. IG = ignorance group; OG = overestimation group; UG = underestimation group; KG = knowledge group.

The mean difference in consumers’ perceived risks was statistically significant. Specifically, OG consumers showed the highest perceived risks (3.94) compared with other groups. This result showed that although OG consumers had higher subjective knowledge, they suspected that GM foods have higher risks.

With regard to perceived benefits, OG consumers felt that GM foods had fewer benefits (2.93) compared with the other three groups. For purchase intention, OG consumers showed the lowest intention (2.42). The purchase intention score of IG consumers was 2.58 and that of UG consumers was 2.62. In contrast to OG consumers’ lowest intention, UG consumers had the second highest intention. The result that OG consumers showed the highest perception of risks and lowest perception of benefits suggests that this group did not take a balanced view of GM foods because of a lack of objective knowledge compared with high subjective knowledge. In this sense, the result of purchase intention can be construed to mean that OG consumers had relatively lower purchase intention than other groups due to their subjective judgment without adequate knowledge.

## Discussion and Conclusion

This study examined consumers’ responses to GM foods regarding risk and benefit perceptions and purchase intention based on their knowledge types. Given that technological advances in the food industry are inevitable for the purpose of sustainable development, understanding how consumers respond to this change is essential for creating a more consumer-oriented environment in the food product market. The major results of this study are as follows.

First, Korean consumers’ level of objective and subjective knowledge is relatively low, similar to that of European and American consumers.^58,59^ In addition, we found that consumers tended to have higher objective knowledge. This result implies that consumers may underestimate their actual knowledge, which may lead them to feel incompetent. These results indicate that most consumers may remain uninformed and even if they have proper knowledge, they might feel helpless due to a lack of subjective knowledge, which undermines their confidence regarding the consumption of GM foods. In that case, consumers may delay or avoid making decisions because they feel anxiety or uncertainty about their purchase consequences.^60^

Second, the result of the multinomial regression showed that education is a significant determinant of imbalanced knowledge groups. Consumers who have more education tend to be classified into the OG; on the contrary, consumers with less education are more likely to be classified into the UG. This result implies that consumers with lower education feel less confident despite high objective knowledge; more educated consumers are overconfident about their knowledge. Moreover, the higher the level of food involvement that consumers have, the more likely they are to be classified into the OG. Consumers who lack education and proper knowledge regarding GM foods may have a distorted perspective about them.^61,62^ Objective knowledge needs to be delivered through possible education programs, especially for consumers who have higher involvement in foods and higher confidence in GM foods but less objective knowledge.

Further, objective knowledge needs to be complemented by a reassuring approach, especially for less educated consumers. Consumers’ media dependency and GM food-related information tendency are significant determinants of their knowledge levels. Specifically, consumers who depend on online media are more likely to be classified into the KG. In other words, if consumers referred to online media more frequently, they had a high level of objective knowledge and felt competent regarding GM foods. In general, educating and empowering consumers by providing useful information is considered a way to help people avoid food safety risks.^63^ The results of this study showed that as the dependency increases on online media to acquire information on GM foods, consumers are more likely to have higher confidence in GM foods based on sufficient actual knowledge. Today, various types of information are delivered immediately and widely through online media, such as internet websites or social media, which can be used to improve consumer knowledge. However, it must be noted that online information may contain not only expert sources of information but also consumer-generated, unsubstantiated content, and therefore, consumers who depend more on online media may shape their attitudes toward GM foods based on confusing, anecdotal, or inaccurate information. Moreover, consumers who have been more exposed to negative information are more likely to overestimate their actual knowledge. This means that one-sided information that leans toward negative tendencies may affect consumers’ beliefs that they have sufficient knowledge without actual objective knowledge. It seems that consumers are more conscious of negative information and may feel seriously threatened by the risks of GM foods, which may make them turn to their subjective belief regarding GM foods rather than to objective information. In this context, science-based information with a balanced focus on the benefits and risks of GM foods needs to be provided through commonly used mass media channels perceived as helpful by consumers.^63^ This would empower them to make decisions by estimating the benefits and risks of GM foods.

Third, consumers’ attitudes and purchase intention were influenced by their knowledge level, because if consumers are uninformed and confused about GM foods, they may have a defensive attitude and hesitate to choose these foods.^2,64^ Consumers’ perceived risks were lowest for the group that underestimated its actual knowledge. In contrast, consumers who overestimated their actual knowledge showed the highest perceived risks. Further, the OG showed the lowest perception of the benefits of GM foods and the lowest purchase intention. By contrast, consumers who have high objective and subjective knowledge were likely to have higher purchase intention toward GM foods. These results imply that consumers who have a high level of subjective knowledge tend to be more conscious about the risks of GM foods and less attentive toward the benefits. Because consumers in this group may simply perceive that they have sufficient knowledge of GM foods, they cannot estimate the risks and benefits of such foods and make a decision based on factual evidence if they are afraid of the related risks.

A challenge for future research is to investigate tailored approaches to these consumers’ knowledge groups to provide information about GM foods effectively. As discussed in this study, these groups have not only different perceived risks and benefits but also different purchase intentions toward GM foods. The determinants of these groups verified in this study showed which factors must be considered when the food industry attempts to shape consumers’ attitudes and purchase intention toward GM foods. Therefore, more specific strategies that can enhance consumers’ acceptance of novel technologies in the food industry according to different consumer knowledge types could be examined in future research. Specifically, delicate and effective communication programs that provide information about GM foods according to consumers’ levels of objective and subjective knowledge could be developed and the effectiveness of communication programs tested empirically. In particular, as today’s media landscape is increasingly based online, where the question of information credibility is raised, the extent to which consumers trust different online information sources needs to be addressed. Further, future studies should also examine how consumers, who are dependent on these online sources, perceive the risks and benefits of GM foods and their intention to accept these foods.

### Policy Implications

The policy implications arising from this study are multifaceted. First, the results of this study showed that both the objective and subjective knowledge of consumers were relatively low. As the low level of objective knowledge indicates that sufficient information has not been delivered to the consumer and/or not accepted by the consumer, it will be important to deliver accurate information from credible organizations to consumers at understandable levels. Further, the uncertainty caused by the disparity between the objective and subjective knowledge on GM foods may increase the unnecessary psychological costs for consumers. Therefore, it is necessary to reduce the imbalance between consumers’ objective knowledge and subjective knowledge through consumer education.

Second, it is possible to increase the efficiency of consumer policy by providing customized education to consumers who have an imbalance between objective and subjective knowledge. Therefore, customized information provision and consumer education should be provided based on a full understanding of the situation of these consumers.

Third, the unconfirmed information on GM foods is provided through various channels, such as the internet websites or social media. However, if this information is inaccurate, consumers are likely to have a distorted view of GM foods. Therefore, there is a need for appropriate government-level regulation of agencies that provide indiscriminate information about GM foods.

The high technical expertise required for GM foods and the controversy over it regarding safety issues make it difficult to leave the development and use of GM food technology completely to the market.^65^ However, most importantly, this is largely attributable to the lack of public trust in government agencies related to GM foods.^66,67^ Therefore, since most countries have established and enforced strict safety management policies at the government level, the general public’s attitude toward GM foods is inevitably affected by the national confidence of the people.^68^ According to the OECD,^69–71^ out of 35 OECD countries in 2015, Korea ranked 26th with 34%, showing relatively low national confidence; however, in 2018 it ranked 25th with 36%. Further, in 2019, it ranked 22nd with 39%, showing that national reliability had gradually increased. Therefore, in order for Korean consumers to have a positive attitude toward GM foods in the future, it is necessary for the Korean government to expand the safety management system for GM foods and secure public understanding and trust in the government.

## Appendix

*The objective knowledge instruments*

**Table:**

Items	True	False	Don’t know
1. GMOs that have drought resistance, herbicide resistance, and pest resistance help to reduce greenhouse gas exhaustion and agricultural chemical use.			
2. GMOs can be cultivated and used only if their safety is examined.			
3. Most of the soybeans and cotton (about 80%) produced all over the world is genetically modified.			
4. Currently, GMOs are not produced in Korea.			
5. GM foods that are currently imported and marketed have been examined for safety.			
6. A labeling system is implemented for GM foods.			
7. A professional organization is operating in Korea that gathers, manages, provides, and communicates information about GMOs and GM foods.			

*The subjective knowledge instrument*

**Table:**

*Item*	Not at all	Don’t know	Know somewhat	Very much
How much do you know about GMOs and GM food?				
